# Relevance of the Glutathione System in Temporal Lobe Epilepsy: Evidence in Human and Experimental Models

**DOI:** 10.1155/2014/759293

**Published:** 2014-11-30

**Authors:** Noemí Cárdenas-Rodríguez, Elvia Coballase-Urrutia, Claudia Pérez-Cruz, Hortencia Montesinos-Correa, Liliana Rivera-Espinosa, Aristides Sampieri, Liliana Carmona-Aparicio

**Affiliations:** ^1^Laboratory of Neurochemistry, National Institute of Pediatrics, 04530 Mexico City, Mexico; ^2^Laboratory of Neuroplasticity and Neurodegeneration, CINVESTAV, 07360 Mexico City, Mexico; ^3^Service of Endocrinology, National Institute of Pediatrics, 04530 Mexico City, Mexico; ^4^Laboratory of Pharmacology, National Institute of Pediatrics, 04530 Mexico City, Mexico; ^5^Department of Comparative Biology, Faculty of Sciences, National Autonomous University of Mexico, 04150 Mexico City, Mexico

## Abstract

Oxidative stress, which is a state of imbalance in the production of reactive oxygen species and nitrogen, is induced by a wide variety of factors. This biochemical state is associated with diseases that are systemic as well as diseases that affect the central nervous system. Epilepsy is a chronic neurological disorder, and temporal lobe epilepsy represents an estimated 40% of all epilepsy cases. Currently, evidence from human and experimental models supports the involvement of oxidative stress during seizures and in the epileptogenesis process. Hence, the aim of this review was to provide information that facilitates the processing of this evidence and investigate the therapeutic impact of the biochemical status for this specific pathology.

## 1. Oxidative Stress: Antioxidant Defense Mechanisms 

Mitochondria are the organelles responsible for most ATP production in nonphotosynthetic organisms. Mitochondria produce energy by oxidizing carbohydrates and fats through the tricarboxylic acid (TCA) cycle and *β*-oxidation, respectively. Electrons from nicotinamide adenine dinucleotide phosphate (NADH) and the reduced form of flavin adenine dinucleotide (FADH_2_), which are produced through the TCA cycle, are transferred to the electron transport chain in the inner mitochondrial membrane. Although the primary function of mitochondria is to produce ATP, they are also critically involved in the control of apoptosis and calcium homeostasis as well as the production and detoxification of reactive oxygen species (ROS) [1].

The physiological levels of ROS can be scavenged by enzymatic (e.g., superoxide dismutase (SOD), glutathione peroxidase (GPx), glutathione reductase (GR), and catalase (CAT)) and nonenzymatic (e.g., vitamin C, vitamin E, carotene, coenzyme Q, melatonin, and reduced forms of glutathione (GSH)) antioxidant defense systems. However, excessive ROS levels caused by increased ROS production or decreased antioxidant defense can lead to oxidative stress [2], which damages proteins, phospholipids, and mitochondrial DNA and results in cell death [3].

Antioxidants can delay or prevent the oxidation of a substrate [4]. The physiological production of ROS in aerobic organisms requires the presence of a defense system to protect against the effects of these oxidative species. This antioxidant defense system has two parts: high molecular weight (antioxidant enzymes, including SOD, CAT, and peroxidases) and low molecular weight (nonenzymatic antioxidants, including vitamins, lipoic acid, uric acid, and GSH) [5].

### 1.1. GSH: Activity of Antioxidant Enzymes

GSH is involved in the activity of the antioxidant enzymes GPx, GR, and GST.

(a) GPx is a selenoenzyme that catalyzes the reduction of two molecules of peroxide using reduced GSH, and it is found in several isoforms [6–9]. Cytosolic GPx (cGPx, GPx-1) is present in the cytosol of all tissues at different concentrations. Plasma GPx (pGPx, GPx-3) occurs in the extracellular fluid of various tissues and in high concentrations in the kidney. Phospholipid GPx (PHGPx, GPx-4) in the membrane and cytosol of various tissues functions as an antioxidant in the cell membrane, and it is abundant in testicles. Gastrointestinal GPx (GI-GPx, GPx-2) is present in the cytosol of the liver and intestinal tract of humans.

(b) The GR enzyme is found in the cytoplasm and has an FAD+ coenzyme at its active site, and it catalyzes the reduction of glutathione disulfide (GSSG) using the coenzyme NADPH [10]. Evidence indicates that NADPH reduces FAD^+^, which transfers two electrons to the disulfide linkage (–S–S–) between two active site cysteine residues. The two –SH groups that are formed then interact with GSSG, reducing it to two GSH molecules. GR maintains the GSH levels in the cell [11].

(c) GSH S-transferase (GST) is an enzyme that is found as cytosolic and microsomal isoforms. Cytosolic GST is divided into four main families (*α*, *μ*, *π*, and *θ*) and four minor families (*ζ*, σ, *κ*, and *ω*), whereas microsomal GST is divided into three families (MGST1, MGST2, and MGST3). Cytosolic GST consists of two identical protein subunits, whereas microsomal GST is a trimer [12–14]. The principal function of this enzyme is the conjugation of GSH with numerous organic compounds; GST can also reduce lipid hydroperoxides, such as GPx, and may detoxify 4-hydroxynonenal (4-HNE), a product of lipid peroxidation.

## 2. Role of GSH in Neurodegenerative Diseases 

Currently, the etiology of neurodegenerative disease lacks an explanation. Many nervous system (NS) disorders are followed by cognitive function deterioration. Among these neurodegenerative processes, a progressive loss of specific neuronal populations is observed, resulting in scenarios in which ROS play a central role. However, the neuronal death that occurs because of trauma, ischemia, inflammatory lesions, excitotoxicity, and excessive ROS may trigger the degenerative process in certain diseases, such as Alzheimer's disease (AD) [15, 16], Parkinson's disease (PD) [15, 17], Huntington's disease (HD) [15, 18, 19], amyotrophic lateral sclerosis (ALS) [20–22], Friedreich's ataxia (FRDA) [23, 24], and epilepsy [25–27]. These diseases manifest a number of complications, including impaired cognition, motor function, and dementia.

The brain is particularly vulnerable to oxidative stress because of its high oxygen consumption; in addition, it contains unsaturated fatty acids that are targets of lipid peroxidation [28–30]. GSH is synthesized in brain cells, and intracellular concentrations have been observed in the range of 0.2 to 10 mM [31]. In 1989, Chen et al. reported GSH levels in different brain regions (i.e., cortex > hippocampus > brainstem) and showed results similar to those reported by Abbott et al. [32, 33]. The GSH level in the cerebrospinal fluid (CSF) is much lower (~5 *μ*M) than in brain tissue [34, 35]. GSH depletion can enhance oxidative stress and may also increase the levels of excitotoxicity molecules, and both actions can initiate cell death in distinct neuronal populations. Similarly, GSH plays multiple roles in the NS, including that of a free radical scavenger, redox modulator of ionotropic receptor activity, and possible neurotransmitter.

The involvement of GSH in neuronal diseases was first described in the neuronal ceroid lipofuscinoses (NCLs), which is known as Batten disease. In an initial study, the formation of pathological “lipopigments” was observed, which was possibly because of an increased rate of peroxidation of polyunsaturated fatty acids [36]. Subsequent research has shown that, in the blood cells of patients with NCLs, the loss of oxidant enzymes is different from that in the controls [37, 38]. The loss of GSH is considered an early change that is responsible for the increased susceptibility to oxidative stress, and it is also associated with aging and leads to neuronal degeneration.

Numerous studies have been conducted to determine the role of GSH in PD, AD, HD, ALS, FRDA, and epilepsy. Pearce et al. were the first to determine that the GSH content in the substantia nigra is significantly lower than in other brain regions [39]. Similar results were obtained in autopsied human brains with PD [40, 41]. In addition, disturbances in brain GSH metabolism may contribute to the development of AD and PD [42–44]. In 2005, Liu et al. showed for the first time that GSH metabolism was regulated differently in male and female AD patients [45].

Furthermore, the reduction of GSH has been reported in the spinal cord in a mouse model of ALS [46]. GSH metabolism disorders might be key risk factors for ALS. Numerous clinical studies have reported an altered redox cycle in ALS patients, including alterations in the synthesis of GPx and activities of other antioxidative enzymes. One study of 35 sporadic ALS patients revealed significantly decreased activities of both GPx and CuZn SOD in the ALS patients compared with that of the controls [47], and additional studies have shown similar results [48, 49]. In postmortem brain samples from 9 patients with sporadic ALS, GPx activity was shown to be reduced in a brain region that is affected in patients with ALS [50].

Reduced GSH and oxidized GSH (GSSG) levels were also observed in various brain areas (substantia nigra, putamen, caudate nucleus, globus pallidus, and cerebral cortex) of patients with HD, and reduced GSSG levels were observed in the caudate nucleus (50%) [51]. In another study, the lipid peroxidation levels were higher and the GSH levels were lower in the plasma of HD patients compared to age- and gender-matched controls [52]. Finally, animal models of HD have shown a significant increase in GSH content in mitochondria isolated from the cortex, striatum [53], hippocampus, and cortex [54].

## 3. Oxidative Stress in Epilepsy: Relevance of GSH

Dysregulation of GSH homeostasis and alterations in GSH-dependent enzyme activities are increasingly implicated in the induction and progression of neuronal diseases, such as epilepsy. It has been widely reported that alterations in the antioxidant system and increases in oxidants are associated with this condition. Epilepsy is one of the most common and serious brain disorders, and it affects at least 50 million people worldwide [55]; in addition, approximately 100 million people will have at least one epileptic seizure during their lifetime.

The brain is particularly susceptible to oxidative stress because it is the organ that utilizes the greatest amount of oxygen within the body. A high content of polyunsaturated fatty acids susceptible to lipid peroxidation, high iron content capable of catalyzing hydroxyl radical formation, and low amounts of CAT increase the susceptibility of the brain to damage from excessive oxidative stress. Oxidative stress is regarded as a possible mechanism in the pathogenesis of epilepsy [56]. Furthermore, persistent seizures have been demonstrated to cause cell damage through increases in oxidative stress [57–61]. As described below, several animal models of epilepsy have consistently found significant increases of ROS after seizures, and important alterations in the antioxidant system have been found in seizing animals and epileptic patients.

Clinical studies have also demonstrated that epileptic patients show a higher lipid peroxidation rate and lower concentrations of vitamins C and A in plasma compared with healthy controls. In one study, epileptic patients treated with phenobarbital and who did not present with convulsions for one year had higher GR levels compared with their pretreated conditions, suggesting that free radicals may be implicated in epilepsy [62]. Other studies have found low levels of selenium (Se) and GPx in patients with epilepsy [63, 64], indicating that impairments in the antioxidant system are highly implicated in seizure generation and recurrence.

### 3.1. Discovering the Relevance of GSH in Epileptic Seizures through the Use of Animal Models

Genetically epilepsy-prone rats (GEPRs) are models of generalized tonic/clonic epilepsy. GEPRs-9 animals develop severe seizures, resulting in hind limb extension [65]. In these animals, hippocampal development is accompanied by oxidative stress, and GEPRs-9 animals cannot compensate for a breakdown in GPx enzymatic activity. These animals show a decrease in GPx and the GSH/GSSG ratio and an increase in GSSG, lipid peroxidation, and protein oxidation, suggesting that the enhanced oxidative burden in GEPR-9s may be caused by their failure to respond to reduced GPx along with the perturbed GSH status in this strain [66].

The administration of systemic of trimethyltin (TMT) in rat models results in a pattern of damage in the CA3 hippocampal region [67] and dentate gyrus [68]. TMT administration generates phenotypes that are similar to phenotypes in certain human epileptic patients, including seizure susceptibility, hyperactivity, and aggression [69]. TMT decreases the ratio of GSH to GSSG, GSH-immunoreactivity, and GPx and GSH protein expression levels in rat hippocampi [70]. However, an elevation in GSH S-transferase (GST) activity has been observed in the hippocampi of mice treated with TMT, suggesting that GST activation may be responsible for the reduced levels of GSH found in the hippocampus [71]. The above data suggest important alterations in the antioxidant system in seizing animals, specifically the GSH defense system. Decreased enzymatic activity is observed after seizures, and if it is restored, seizures can be reduced.

### 3.2. Antiepileptic Drugs and Oxidative Stress in Epileptic Patients

Several studies have demonstrated that antiepileptic drugs (AEDs) may impair the antioxidant defense system and induce or exacerbate oxidative injury in epileptic patients. Several first-choice AEDs for epileptic syndromes, such as valproic acid, carbamazepine, phenytoin, and phenobarbital, increase lipid peroxidation and nucleic acid oxidation in the blood [72–78]. In addition, phenytoin decreases the GSH serum levels in adult epileptic patients, carbamazepine reduces GPx levels, and phenobarbital decreases SOD and GPx levels in the erythrocytes of adult patients [76]. Carbamazepine has also been shown to induce fewer disturbances to antioxidant and lipid peroxidation relative to valproic acid [74, 75] or phenytoin [76].

Alterations in the antioxidant system induced by AED therapy can be explained by the metabolism of AED into reactive epoxide intermediates, which bind covalently to bimolecular and induce structural and functional impairments [79]. AED treatment has also been associated with cognitive decline [80, 81], and high doses increase the risk of cognitive side effects [82, 83]. Therefore, researchers are now focusing on developing add-on treatments for AED therapies to counteract the cognitive decline induced by antiepileptics and seizures. In various studies, a beneficial effect of melatonin as an add-on therapy for epileptic children was described in a series of clinical trials [84–87]. In addition, the add-on melatonin treatment was reported to elevate GR and GPx activities in the blood of epileptic children receiving valproic acid or carbamazepine monotherapy [85, 86]. These authors also observed that melatonin improved sleep patterns, behavior, attention, cognition, and memory function in epileptic children receiving valproic acid monotherapy, which may reflect a neuroprotective effect of melatonin against the deleterious effects of epilepsy and AED therapy [85, 86].

In another study, topiramate (TPM), a new antiepileptic compound that acts by inhibiting voltage-gated sodium and calcium channels, blocking glutamate AMPA/kainite receptors and enhancing GABA_A_ receptor-mediated chloride, was tested as a neuroprotective agent [88]. TPM therapy in combination with selenium supplementation (TPM + Se) decreases erythrocyte lipid peroxidation and increases GSH and GSH-GPx along with plasma vitamins A and C in refractory epileptic patients [89]. Moreover, in pentylenetetrazol- (PTZ-) treated rats, TPM and TPM + Se decreased lipid peroxidation in plasma and erythrocytes and increased GSH levels compared with that of the control group [90]. Therefore, TPM appears to be a promising new AED that not only ameliorates epileptic seizures but also can shield the brain from the damaging effects of oxidative stress.

## 4. GSH in Temporal Lobe Epilepsy

The evidence for GSH involvement in epilepsy is well known, and we have provided evidence of such involvement in experimental models (Table 1) as well as in humans (Table 2).

## 5. Potential Use of Antioxidants against Seizures and Their Epileptogenic Impact on the GSH 

Current research has suggested that antioxidant compounds may provide some level of neuroprotection against the neurotoxicity of seizures at the cellular level. Epigallocatechin-3-gallate (EGCG) is the main polyphenol of green tea and possesses important antioxidant, anti-inflammatory, and antiapoptotic properties [91–93]. Recently, it has been demonstrated that EGCG suppresses the progression of kindling and ameliorates the cognitive impairment in PTZ-kindled rats [94]. Moreover, EGCG treatment restores the oxidative stress induced by PTZ-kindling by increasing brain GSH levels [94].

During the last decade, curcumin has been the focus of attention in the field of antioxidant and anti-inflammatory research because of its promising effects in several diseases. Curcumin is a low molecular weight polyphenol found in the dried ground rhizome of the perennial herb* Curcuma longa* [95]. Recently, it has been tested for its neuroprotective properties in neurodegenerative diseases, such as AD [96], and it has also been tested as an anticonvulsant and cognitive enhancer. As previously mentioned, AED treatment has been associated with cognitive decline, and Reeta et al. investigated the neuroprotective effect of curcumin against phenytoin-induced cognitive impairment in rats [97]. These authors observed that curcumin produced a dose-dependent reduction of phenytoin-induced brain malondialdehyde (MDA) and dose-dependent increase in GSH brain levels. These effects were accompanied by positive effects on retention transfer latency in an elevated plus maze test and the passive avoidance paradigm. Moreover, curcumin alone (administered for 21 days) decreased oxidative stress levels, which was indicated by a significant decrease and increase in brain MDA and GSH levels, respectively [97]. Several studies have focused on the antiepileptic actions of curcumin, which has also been shown to have anticonvulsant properties against seizures induced by KA [98, 99] and FeCl_3_ [100] and an ability to elevate the seizure threshold in the maximal electroshock model [101]. In addition, curcumin can reduce the incidence of status epilepticus induced by pilocarpine [102] and inhibits amygdala-kindled seizures in rats [103]. Moreover, curcumin restores pilocarpine-induced decreases in hippocampal GSH and SOD levels [102], indicating that enhancing the antioxidant system may be a potential strategy to combat epileptic seizures.

## 6. Other Antiepileptic Therapies That Regulate GSH Levels

The classic ketogenic diet (KD) is a high-fat/low-carbohydrate diet (most often in a 4 : 1 fat : nonfat ratio) that is used to treat intractable seizures in children and adolescents. Our understanding of the metabolic effects of a KD originates from the pioneering work of Cahill and colleagues in the 1960s, but the importance of these diets from a clinical perspective was acknowledged in the early 1920s with their successful use in the treatment of epilepsy.

Several theories have focused on the potential protective role of ketone bodies that accumulate during ketosis; recently, an alteration in mitochondria bioenergetics caused by the application of a KD has been suggested. GSH levels are an important indicator of mitochondrial and cellular health, and as mentioned above, mitochondrial dysfunction has been implicated in seizures and epilepsy.

Mice fed a KD for 10–12 days have been shown to produce less ROS [104]. In addition, rats maintained on a KD for at least 4 weeks have been found to have significantly more mitochondria in their hippocampi compared with that of controls, suggesting that mitochondrial biogenesis is stimulated by the consumption of a KD [105]. Several markers of redox status, such as increases in the GSH/GSSG ratio, have shown improvement in the hippocampi of rats fed a KD [106]. Furthermore, the increased activity of glutamate cysteine ligase (GCL), the rate-limiting enzyme in GSH biosynthesis, has been shown in the hippocampi of rats fed a KD. In addition, rats fed a KD produce less H_2_O_2_ than controls, suggesting functional improvements as a result of consuming a KD. It has been suggested that activation of the Nrf2 transcription factor plays an important role in the enhanced biosynthesis of GSH by KDs [107]. Although the exact mechanism by which a KD provides neuroprotection remains unclear, the previously mentioned results suggest that restoring the antioxidant system may be linked to the anticonvulsant properties of a KD.

## Figures and Tables

**Table 1:** Evidence of the GSH system in experimental epilepsy models.

Model	Procedure model	Observations	References
Electrical implants in male Sprague-Dawley rats (300–500 g)	Insulated stainless steel electrodes were implanted in the left dentate gyrus and angular bundle. During the experiments, video-EEG was continuously recorded (24 h/day) until the animals were sacrificed. Plasma was used by biochemical determinations.	The glutathione PEGylated (GSH-PEG) liposomal methylprednisolone (MP) treatment did not have any effect on SE duration and subsequent seizure development. Both the GSH-PEG liposomal MP-treated and vehicle-treated rats developed spontaneous seizures, indicating that GSH-PEG liposomal MP could not prevent epileptogenesis.	[108]

Hippocampal glutamine synthetase deficiency by continuous microinfusion of methionine sulfoximine (MSO) in male Sprague-Dawley rats (180–220 g)	An osmotic pump was introduced through a burr hole in the skull and then into the right hippocampus. The pumps were filled with MSO to achieve the following drug delivery rates: 2.5, 1.25, and 0.625 *µ*g/h for approximately 28 days. Separate pumps were filled with saline (0.9% NaCl) as a control. For the GSH determination, the hippocampi were isolated. GSH was measured using the spectrophotometric method with 5-thio-2-nitrobenzoic acid in a reaction coupled with GR.	Recurrent behavioral seizures occurred with all doses of MSO. The intrahippocampal infusion of MSO was associated with a dose-dependent loss of neurons in the hippocampal formation and nearby brain areas. No decrease in hippocampal GSH was observed in the lower-dosed animals (0.625 *µ*g/h), whereas a 21% decrease was observed in the higher-dosed animals (2.5 *µ*g/h) 10 days after the onset of MSO infusion.	[109]

Lithium-pilocarpine in male Sprague-Dawley rats (260–300 g)	Lithium chloride (LiCl) (127 mg/kg) was injected intraperitoneally (i.p.) into both the experimental and control groups. Status epilepticus (SE) was induced by a subcutaneous injection of pilocarpine hydrochloride (25 mg/kg) 20 h after the LiCl treatment. For the GSH determination, the hippocampus, dentate gyrus, amygdala, entorhinal, piriform cortices (hippocampal formation), cerebral cortex, and cerebellum were removed and evaluated by high-performance liquid chromatography (HPLC).	The concentration of GSH was decreased in the hippocampal formation (22.6%) and cerebellum (6%) in the epileptic rats.	[110]

Pilocarpine in 7- to 8-week-old male CD1 mice (25–40 g)	A single dose of pilocarpine was administered (330–345 mg/kg subcutaneously). All determinations with pilocarpine and controls were realized within 3.5–4 weeks after treatment, and the cerebral cortices, HF, and blood samples were obtained. The GSH levels were measured by HPLC.	The level of GSH was significantly decreased (18%) in the hippocampal formation, whereas it was not significantly altered in the cortex in the pilocarpine mice.	[111]

Pilocarpine in 2-month-old male Wistar rats (250–280 g)	The control animals received 0.9% i.p. saline, and in the experimental group, the animals were treated with a dose of pilocarpine hydrochloride (400 mg/kg, i.p.). To determine the lipid peroxidation level, nitrite content, GSH concentration, and SOD and CAT activities, the rats (pilocarpine and control groups) were sacrificed 24 h after the treatment, and the brains were dissected on ice to remove the frontal cortex and striatum.	After pilocarpine-induced SE, significant increases (i.e., 47 and 59%) in the thiobarbituric acid reactive substance (TBARS) levels in the striatum and frontal cortex were observed. Marked increases were presented in nitrite content: 49 and 73% in the striatum and frontal cortex, respectively; the GSH concentrations decreased by 54 and 58% in the striatum and frontal cortex, respectively; the SOD in frontal cortex was verified by its increase of 24% after the seizures; and CAT increases of 39 and 49% were observed in the striatum and frontal cortex, respectively.	[112]

Pilocarpine-lithium in 80- to 90-day-old male and female Wistar rats	SE was induced by administering pilocarpine hydrochloride (30 mg/kg i.p.) 22 h after LiCl (127 mg/kg i.p.). SE was interrupted after 2 h, and the rats were sacrificed 24 h later. The piriform and entorhinal cortices, temporal neocortex, thalamus, and hippocampus were dissected. Neurochemical determinations were performed using spectrophotometric methods: lipid peroxidation was analyzed by measuring the TBARS levels; SOD activity was analyzed with the xanthine-xanthine oxidase system, and GPx was analyzed by reducing the cumene hydroperoxide using GSH as a reducing agent.	The TBARS levels in all of the examined structures were significantly higher in the rats with SE: approximately 41% higher in the piriform and entorhinal cortices; 22% higher in the temporal neocortex; 25.7% higher in the thalamus and 15% higher in the hippocampus. SOD activities were significantly higher in the rats with SE in the piriform and entorhinal cortices (11.7%) and temporal neocortex (19.7%). The GPx activities were significantly higher in the animals with SE in the piriform and entorhinal cortices (22.1%) and thalamus (8.9%). The authors did not observe significant sex-treatment interactions in the results in any of the investigated brain regions.	[113]

Pilocarpine in male Wistar rats (250–350 g)	The experimental group was injected with pilocarpine (350 mg/kg i.p.), and the control rats were injected with a physiological salt solution. The rats were sacrificed by decapitation 2 h after drug administration, and the cortical regions were removed. Neurochemical determinations were performed by spectrophotometric methods: lipid peroxidation was analyzed by measuring the oxidative marker malondialdehyde (MDA); SOD activity was measured with the xanthine/xanthine oxidase system; GPx was measured with H_2_O_2_ as the substrate and GR and NADPH as the enzymatic and nonenzymatic indicators, respectively; CAT activity was measured by H_2_O_2_ decomposition and GR and NADPH as the enzymatic and nonenzymatic indicators, respectively. The mRNA expression of the antioxidant enzymes was determined by real-time RT-PCR.	Pilocarpine increased the MDA levels (64%). All enzymatic activities were measured, and CAT, GPx, and SOD significantly increased in response to pilocarpine (28%, 28%, and 21%, resp.). The GPx gene expression significantly increased in the pilocarpine group (1.47-fold), and the Mn-SOD expression also significantly increased (1.33-fold). The CAT expression was unchanged.	[114]

Kainite in male Sprague-Dawley rats (300–350 g)	The rats were subcutaneously administered saline or 11 mg/kg kainite. The rats were sacrificed after 1 min of carbon dioxide inhalation and then were immediately decapitated at 8 h, 24 h, 48 h, 1 week, 3 weeks, and 6 weeks after injection to determine the acute, latent, and chronic periods of epileptogenesis. The hippocampal tissue was prepared for biochemical analysis. GSH and GSSG were determined by HPLC.	Whole hippocampal tissue GSH decreased during the acute, latent, and chronic stages of the experimental temporal lobe epilepsy (TLE). Hippocampal tissue GSSG levels increased substantially at 48 h after kainate treatment. Acute GSSG was increased at the 8 and 24 h time points. During the latent period, GSSG was elevated from 1 to 6 weeks after the kainite treatment. The GSH/GSSG ratio was significantly decreased in the kainate treatment groups from 24 h through 6 weeks.	[115]

**Table 2:** Evidence of the GSH system in epilepsy patients.

Patients characteristics	Criteria	Methods	Observations	Reference
Nine patients (5 males and 4 females, age 37 ± 6 years) diagnosed with refractory TLE. Thirty-two healthy individuals without any drug treatments were used as controls. The age range and gender distribution of the control group were similar to the patient group (18 males and 14 females, age 37 ± 9 years). The patients were recruited from the National Center of Medical Genetics in Cuba.	*Inclusion criteria* Patients with refractory TLE, seizures for at least the last 2 years, and a minimum of two monthly complex partial seizures; failure of two major antiepileptic drugs and two monotherapy cycles and at least one polytherapy. *Exclusion criteria* Serious systemic illnesses, idiopathic epilepsy, simple partial seizures as the only type of seizure, being unresponsive to treatment, or active psychotic illness. Patients retained their habitual drug treatments (drugs and doses) during the entire study. Serum samples from 32 healthy individuals without any drug treatments were used as the controls. The age range and gender distribution of the control group were similar to those of the patient group (18 males and 14 females, age 37 ± 9 years).	Neurochemical determinations were performed in serum in the control group and lobectomy group using spectrophotometric methods: SOD activity was measured by the ability to inhibit autoxidation of pyrogallol; CAT activity was measured by H_2_O_2_ decomposition; and GPx was measured with H_2_O_2_ as the cumene hydroperoxide and GR and NADPH as the enzymatic and nonenzymatic indicators. Lipid peroxidation was analyzed with the MDA adduct. An anterior temporal lobectomy was performed after the presurgical evaluation. Determinations were performed at the presurgery stage as well as at 1, 6, 12, and 24 months after surgery.	The CAT enzymatic activity in the patients showed the greatest dispersion among the antioxidant enzymes and was not significantly different from that in the controls except at 6 months after surgery. The SOD activity in the patients was significantly different from the control group at each studied stage. The postsurgery stages were not significantly different from the presurgery phase. Statistically significant differences in GPx were observed when comparing the presurgery stage and the 1-month and 6-month postsurgery stages with the control group. Statistical significance for MDA occurred when comparing the presurgery, 1-month, 6-month, and 1-year stages with the control group. A tendency was observed toward normalization, with significantly different MDA levels at 1-year and 2-year postsurgery in terms of the presurgery values. In the group of patients with TLE, a negative correlation between GPx and MDA was observed at each studied stage. This correlation was also statistically significant for the control group.	[116]

Twelve patients diagnosed with intractable TLE were recruited from Hacettepe University (6 males and 6 females, 32 ± 11 years of age). The control was a male traffic accident victim. The subject was 35 years old with no known history of any central nervous system diseases and in good health.		GPx gene expression was analyzed by RT-PCR in hippocampectomy specimens.	GPx exhibited an upregulation of 2.3-fold.	[117]

Nineteen patients with epilepsy (12 males and 7 females, 32.7 ± 10.2 years of age). Eight healthy controls (3 males and 5 females, 28.4 ± 10.7 years of age). Eighteen patients with temporal lobe epilepsy (TLE). The patients were recruited from those admitted to the Department of Epileptology at the University Hospital in Zurich.	*Inclusion criteria* Focal epilepsy with an epileptogenic focus defined by an EEG with (*n* = 12 patients) or without (*n* = 7 patients) active epilepsy. The patients with active epilepsy still had seizures at the time of the examination; the patients without active epilepsy were seizure-free at the time of the examination for several months because of a surgical intervention, but they had previously experienced regular epileptic seizures. *Exclusion criteria* Contraindications for MR examination.	The GSH determination was performed by ^1^H-MRS.	Compared to the controls, there was a significant reduction (i.e., approximately 35%) of the mean GSH/water ratio in the parietooccipital regions of the patients. This reduction was found in the brain regions distal to the epileptogenic focus in structurally normal-appearing tissue in all but one patient with an epileptogenic focus in the parietal lobe. The mean GSH/water ratio was not different between the hemisphere containing the epileptogenic focus and the hemisphere without the focus.	[118]

## Abbreviations

AEDs: Antiepileptic drugs
CAT: Catalase
EGCG: Epigallocatechin-3-gallate
GEPRs: Genetically epilepsy-prone rats
GCL: Glutamate cysteine ligase
GPx: Glutathione peroxidase
GR: Glutathione reductase
GSH: Reduced form of glutathione
GSSG: Oxidized form of glutathione
GST: Glutathione S-transferase
H_2_O_2_: Hydrogen peroxide
KA: Kainic acid
KD: Ketogenic diet
${\text{HO}}^{∙}$: Hydroxyl radical
PTZ: Pentylenetetrazol
ROS: Reactive oxygen species
SOD: Superoxide dismutase
Se: Selenium
TCA: Tricarboxylic acid cycle
TLE: Temporal lobe epilepsy
TMT: Trimethyltin
TPM: Topiramate.
